# Retinyl esters form lipid droplets independently of triacylglycerol and seipin

**DOI:** 10.1083/jcb.202011071

**Published:** 2021-07-29

**Authors:** Martijn R. Molenaar, Kamlesh K. Yadav, Alexandre Toulmay, Tsjerk A. Wassenaar, Muriel C. Mari, Lucie Caillon, Aymeric Chorlay, Ivan E. Lukmantara, Maya W. Haaker, Richard W. Wubbolts, Martin Houweling, Arie Bas Vaandrager, Xavier Prieur, Fulvio Reggiori, Vineet Choudhary, Hongyuan Yang, Roger Schneiter, Abdou Rachid Thiam, William A. Prinz, J. Bernd Helms

**Affiliations:** 1 Department of Biomolecular Health Sciences, Faculty of Veterinary Medicine, Utrecht University, Utrecht, Netherlands; 2 Groningen Biomolecular Sciences and Biotechnology Institute, Zernike Institute for Advanced Materials, University of Groningen, Groningen, Netherlands; 3 National Institute of Diabetes and Digestive and Kidney Diseases, National Institutes of Health, Bethesda, MD; 4 Department of Biomedical Sciences of Cells and Systems, University of Groningen, University Medical Center Groningen, Groningen, Netherlands; 5 Laboratoire de Physique Statistique, Ecole Normale Supérieure, Paris Sciences et Lettres Research University, Sorbonne Université, Université Pierre-et-Marie-Curie Université Paris 06, Université Paris Diderot, Centre national de la recherche scientifique, Paris, France; 6 Department of Biology, University of Fribourg, Fribourg, Switzerland; 7 Université de Nantes, Centre national de la recherche scientifique, Institut national de la santé et de la recherche médicale, l’institut du thorax, Nantes, France; 8 School of Biotechnology and Biomolecular Sciences, University of New South Wales, Sydney, New South Wales, Australia

## Abstract

Lipid droplets store neutral lipids, primarily triacylglycerol and steryl esters. Seipin plays a role in lipid droplet biogenesis and is thought to determine the site of lipid droplet biogenesis and the size of newly formed lipid droplets. Here we show a seipin-independent pathway of lipid droplet biogenesis. In silico and in vitro experiments reveal that retinyl esters have the intrinsic propensity to sequester and nucleate in lipid bilayers. Production of retinyl esters in mammalian and yeast cells that do not normally produce retinyl esters causes the formation of lipid droplets, even in a yeast strain that produces only retinyl esters and no other neutral lipids. Seipin does not determine the size or biogenesis site of lipid droplets composed of only retinyl esters or steryl esters. These findings indicate that the role of seipin in lipid droplet biogenesis depends on the type of neutral lipid stored in forming droplets.

## Teaser

Molenaar et al. show that seipin does not play a role in the biogenesis of lipid droplets composed mostly or only of the neutral lipid retinyl ester, even in cells that produce triacylglycerols.

## Introduction

Lipid droplets (LDs) form a ubiquitous class of organelles that store neutral lipids for a multitude of functions. Defects in LD synthesis are linked to a range of diseases (Hashemi and Goodman, 2015; Pol et al., 2014; Thiam and Beller, 2017; Walther and Farese, 2012).

The mechanism of LD biogenesis is incompletely understood. In the most prevalent view, LD formation is primarily driven by triacylglycerol (TAG) synthesis at the ER (Choudhary et al., 2015; Thiam and Forêt, 2016; Walther et al., 2017).

Several proteins and lipids have been identified in the regulation of LD biogenesis. Seipin is an evolutionarily conserved ER integral membrane that forms large, ringlike oligomers and is found in ER foci that are often in contact with nascent LDs. Cells lacking seipin have aberrant LDs, either clusters of small LDs or LDs that are much larger than normal (Binns et al., 2010; Fei et al., 2008; Grippa et al., 2015; Joshi et al., 2018; Salo and Ikonen, 2019; Sui et al., 2018; Szymanski et al., 2007; Wang et al., 2016; Yan et al., 2018). Seipin has also been suggested to determine the site of LD biogenesis in the ER (Chung et al., 2019; Salo et al., 2019), to regulate the flow of neutral lipids and proteins from the ER to nascent LDs at ER–LD contacts (Grippa et al., 2015; Salo et al., 2019; Wang et al., 2014), and to regulate lipid metabolism at LD biogenesis sites (Renne et al., 2020). Recently, seipin was shown to trap TAGs in the ER bilayer via luminal hydrophobic helices (Prasanna et al., 2021; Zoni et al., 2021).

**Figure 1.**
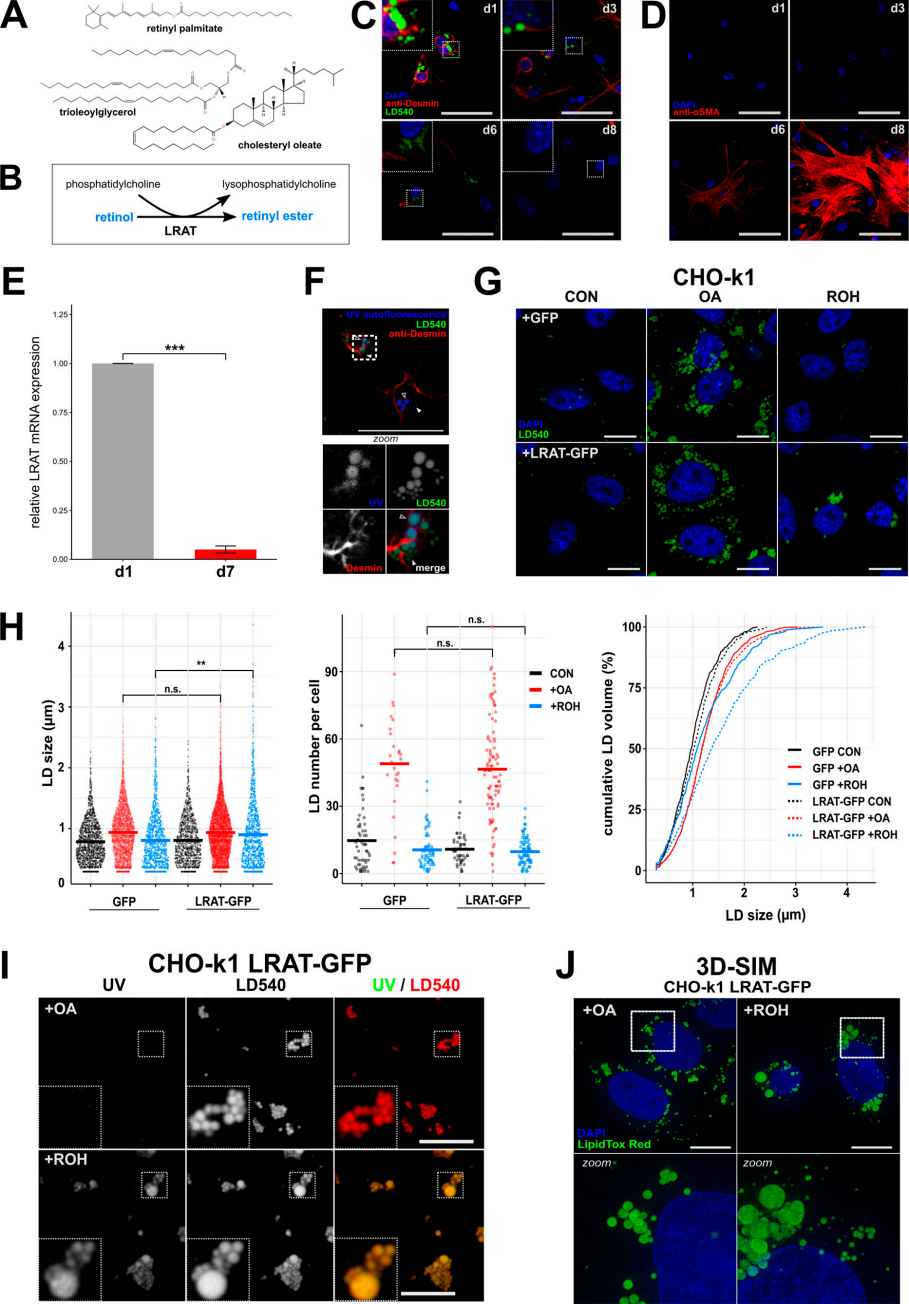
**LDs containing REs have a distinct morphology.** **(A)** Chemical structures of the neutral lipids RP, cholesteryl oleate, and TOG. **(B)** Reaction mechanism of LRAT. **(C and D)** Confocal microscopy of murine HSCs (mHSCs) cultured for 1, 3, 6, or 8 d and stained with DAPI (blue), LD540 (green), and anti-desmin (red, an HSC marker; C), or DAPI (blue) and anti–α-smooth muscle actin (red, a marker of activated HCSs; D). Scale bars indicate 50 µm. Zoomed regions are 16.4 µm wide. **(E)** Relative expression of LRAT mRNA in mHSCs after 1 or 7 d of culture by quantitative PCR. Bar plot indicates mean ± SEM of three animals. Statistical significance was determined by two-tailed paired Welch’s *t* test. **(F)** Confocal microscopy of mHSCs 2 h after isolation showing UV autofluorescence (blue), LD540 (green), and anti-desmin (red). Bottom panel is a zoomed inset of the area surrounded by dotted lines in the main panel. Closed triangles indicate UV^─^ LDs; open triangles indicate UV^+^ LDs. Scale bars indicate 50 µm. Zoomed regions are 14.5 µm wide. **(G–I)** Confocal microscopy of CHO-k1 cells expressing GFP or LRAT-GFP incubated overnight in the presence or absence of 20 µM ROH or 200 µM OA. Imaged channels are DAPI (blue) and LD540 (green; G) or UV autofluorescence (left) and LD540 (middle; I). Scale bars indicate 10 µm. Zoomed regions are 5 µm wide. CON, control. **(H)** Quantification of G showing LD size (left panel), LD number per cell (middle panel), and cumulative LD volume by LD size (right panel). Conditions are colored in black (control), red (200 µM OA), and blue (20 µM ROH; in the respective conditions, LDs counted were *n* = 1,436, 4,064, 1,127, 1,095, 7,373, and 1,132, respectively; cells counted were *n* = 55, 28, 65, 47, 93, and 82, respectively). **(J)** Full projections of 3D-SIM images of CHO-k1 cells expressing LRAT-GFP incubated overnight in the presence 20 µM ROH or 200 µM OA. Bottom panels are zoomed insets (10.8 µm wide) of areas surrounded by dotted lines in the top panels. Scale bars indicate 10 µm. **, P < 0.01; ***, P < 0.001.

The abundant presence of large LDs is a hallmark of hepatic stellate cells (HSCs) in normal liver. HSCs are specialized in the storage of retinol (ROH; vitamin A) as retinyl esters (REs). After liver injury, HSCs lose their characteristic LDs and transdifferentiate into myofibroblasts (Blaner et al., 2009; Friedman, 2008). Recent research shows the presence of two types of LDs: so-called preexisting original LDs with relatively slow turnover and rapidly recycling LDs that transiently appear during activation of HSCs (Testerink et al., 2012; Tuohetahuntila et al., 2016; Ajat et al., 2017; Molenaar et al., 2017). Whereas synthesis and breakdown of TAGs in rapidly recycling LDs are mediated by DAG *O*-acyltransferase 1 (DGAT1) and adipose triglyceride lipase (Tuohetahuntila et al., 2016), less is known about the turnover of preexisting LDs. Inhibition of DGAT1 does not affect the dynamics of the preexisting LDs, nor does it affect the synthesis of REs in isolated primary HSCs (Ajat et al., 2017; Tuohetahuntila et al., 2016). HSCs contain the enzyme lecithin:retinol acyltransferase (LRAT) that catalyzes a transesterification reaction between phosphatidylcholine (PC) and all-trans-ROH to form all-trans-RE (Fig. 1, A and B; Golczak et al., 2012; Ruiz and Bok, 2010). As LRAT is the main contributor to RE storage in the liver (Liu and Gudas, 2005; O’Byrne et al., 2005), we investigated the possibility that LRAT-mediated RE synthesis can drive the generation of LDs in HSCs.

## Results and discussion

### LRAT expression generates UV-positive LDs

Primary and quiescent HSCs spontaneously transdifferentiate into activated HSCs (myofibroblasts) ex vivo upon isolation and subsequent culture, resulting in LD disappearance (Blaner et al., 2009; Friedman, 2008; Fig. 1, C and D). In addition, LRAT expression decreases (Blaner et al., 2009; Kluwe et al., 2011; Fig. 1 E). Using the autofluorescent properties of REs, we observed two populations of LDs in quiescent HSCs: large UV^+^LD540^+^ structures containing REs and UV^−^LD540^+^ structures, lacking REs, with smaller diameters (Fig. 1 F).

**Figure S1.**
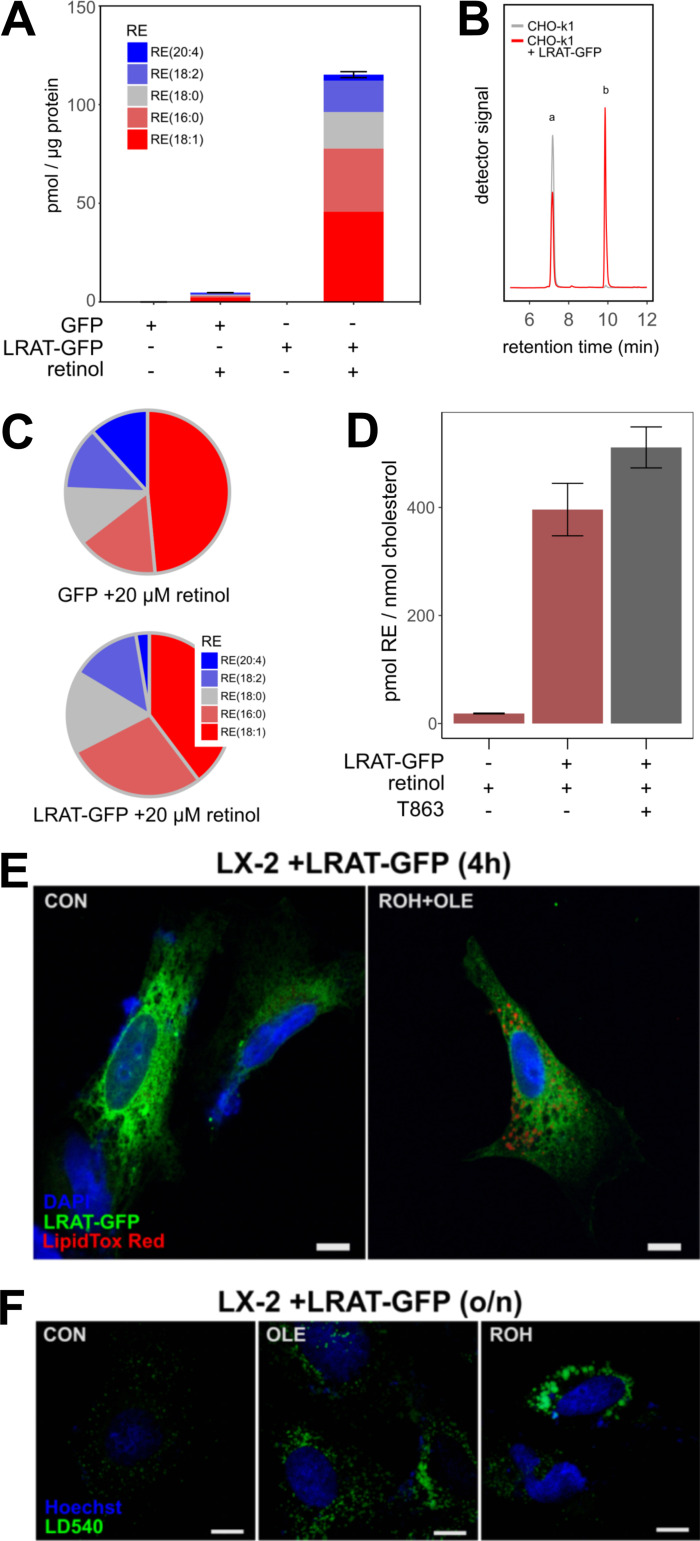
**Quantification of RE content. (A)** CHO-k1 cell lines expressing GFP or LRAT-GFP were incubated in the presence or absence of 20 µM ROH. RE species were analyzed by LC-MS/MS. Amounts are expressed as pmol RE per μg protein (mean ± SD of a representative experiment). **(B)** Chromatogram showing in vitro LRAT activity of CHO-k1 homogenates with (red line) and without (gray line) expression of LRAT-GFP. After incubation with PC (7:0/7:0) and 10 µM ROH, ROH (peak a) and RE (7:0; peak b) were measured by LC-MS/MS. Multiple reaction monitoring transition 269/93 (retinoid backbones) is shown. **(C)** Relative contribution of RE species that were synthesized under the conditions described in A. **(D)** CHO-k1 cell lines with or without expression of LRAT-GFP were incubated overnight with 20 µM ROH in the presence or absence of 10 µM DGAT1 inhibitor T863. Total RE (m/z 269) and free cholesterol (m/z 369) were analyzed by LC-MS. Amounts are expressed as pmol RE per nmol cholesterol (mean ± SD of a representative experiment). **(E)** Confocal microscopy of transiently transfected LX-2 cells expressing LRAT-GFP (green) costained with DAPI (blue) and LipidTOX Red (red). Cells were incubated without (left) or with (right) 20 µM ROH and 200 µM OA for 4 h. OLE, oleic acid. **(F)** Confocal microscopy of transiently transfected LX-2 cells expressing LRAT-GFP stained with DAPI (blue) and LD540 (green). Cells were incubated without (left) or with 200 µM OA (middle panel) or 20 µM ROH (right panel) overnight. Scale bars indicate 10 µm.

To understand the role of LRAT in LD biology, we stably transfected CHO-k1 cells with a plasmid carrying LRAT-GFP. Lipidomic analysis of CHO cells expressing LRAT confirmed RE synthesis only after addition of ROH (Fig. S1 A). In the absence of LRAT (Fig. S1 B), CHO-k1 cells can synthesize REs using a different mechanism involving DGAT1 (Ajat et al., 2017; Orland et al., 2005). This reaction occurs with much lower efficiency and results in a different species profile of REs (Fig. S1 C). Inhibition of DGAT1 activity in CHO-k1 cells expressing LRAT-GFP showed no inhibition of RE synthesis, which confirms that LRAT is the primary RE-synthesizing enzyme (Fig. S1 D). Quantification of LD size and number showed a significant increase of the mean LD size of cells transfected with LRAT-GFP and incubated with ROH (Fig. 1 H, left panel), while no difference in LD number was measured (Fig. 1 H, middle and right panels). For RE^+^ LDs, the top 25% of total LD volume is carried by LDs >2 µm (for TAG^+^ LDs, this is 1.5 µm). In contrast to oleic acid (OA)-stimulated LDs, ROH-induced LDs also exhibited UV autofluorescence (Fig. 1 I). Similar results were obtained after transfection of human HSC-derived cell line LX-2 with LRAT (Fig. S1, E and F). By superresolution microscopy (3D structured illumination microscopy [3D-SIM]), we observed both small and large LDs in LRAT-dependent LD synthesis and with a clustered appearance (Fig. 1 J). In the presence of OA, the LDs displayed a more homogeneous size distribution of relatively small LDs dispersed throughout the cell (Fig. 1 J and Videos 1 and 2).

**Video 1.** **3D reconstruction of LDs in LRAT-GFP.** Reconstructions by 3D-SIM of LRAT-GFP–expressing CHO-k1 cells incubated with 200 µM OA, showing DAPI (blue) and LipidTOX Red (green). Frame rate is 24 frames/s.

**Video 2.** **3D reconstruction of LDs in LRAT-GFP.** Reconstructions by 3D-SIM of LRAT-GFP–expressing CHO-k1 cells incubated with 20 µM ROH, showing DAPI (blue) and LipidTOX Red (green). Frame rate is 24 frames/s.

### LRAT-mediated LD formation is independent of TAG synthesis

**Figure 2.**
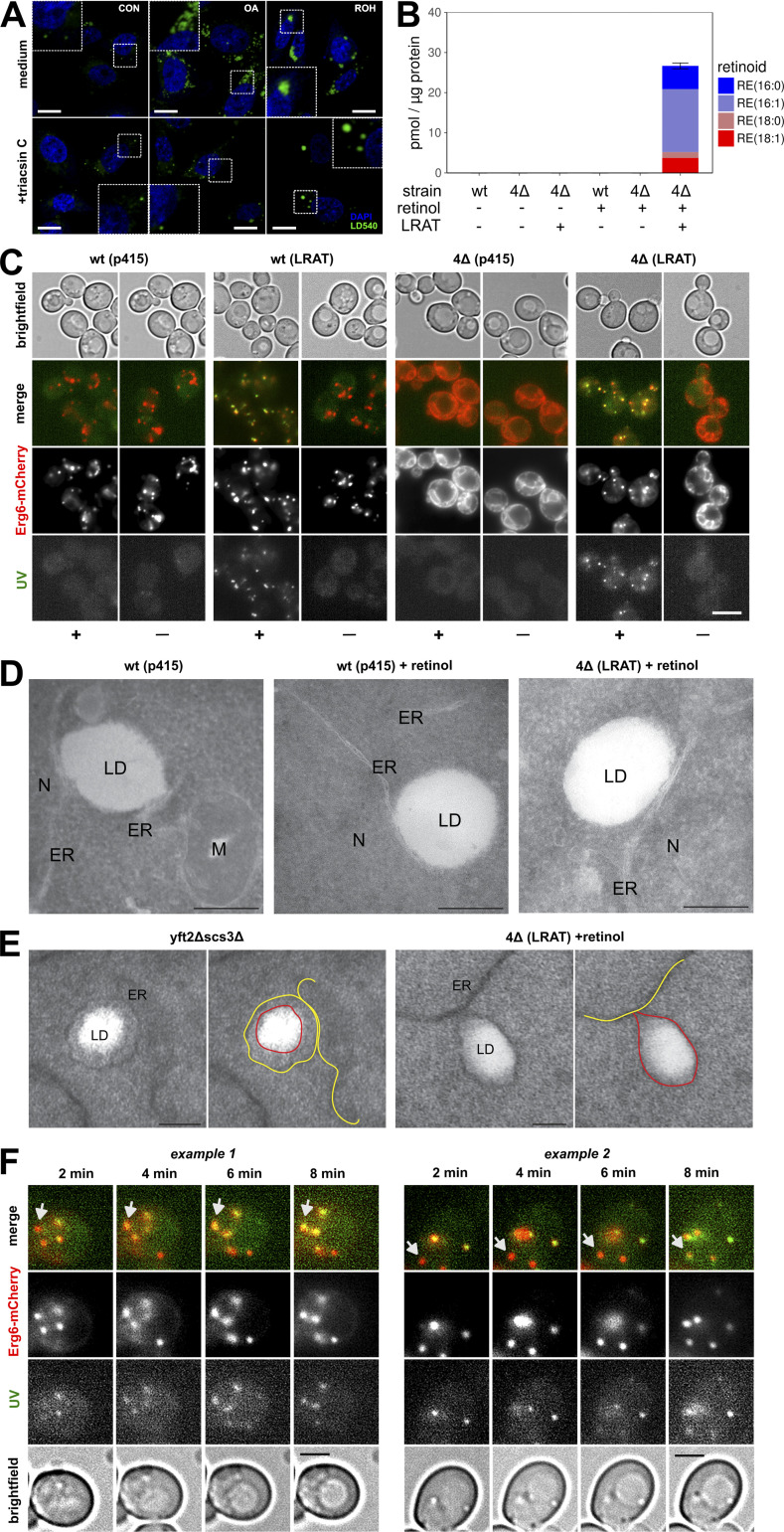
**LRAT-mediated LD formation in the absence of preexisting LDs.** **(A)** Confocal microscopy of CHO-k1 cells expressing LRAT-GFP. After preincubation with medium containing 1% FBS, cells were incubated overnight with or without 200 µM OA or 20 µM ROH in the presence or absence of 1 µg/ml triacsin C. Cells were stained with DAPI (blue) and LD540 (green). Scale bars indicate 10 µm (zoomed regions are 10 µm wide). CON, control. **(B)** Quantification of predominant RE species by LC-MS/MS of WT and 4Δ yeast cells expressing LRAT 2 h after incubation in the presence or absence of 2 mM ROH. Amounts are expressed in pmol RE per μg protein. Bar plot indicates mean ± SD. **(C)** Widefield microscopy of Erg6-mCherry expressing WT and 4Δ yeast cells, with or without expressing LRAT, 2 h after incubation with or without 2 mM ROH (− or +). Images of UV autofluorescence (green), Erg6-mCherry (red), and brightfield were taken. Scale bars indicate 5 µm. **(D)** EM of WT and 4Δ yeast cells, with or without expressing LRAT, incubated with or without 2 mM ROH. M, mitochondria; N, nucleus. Scale bars indicate 250 nm. **(E)** EM of *yft2Δscs3Δ* and 4Δ yeast cells expressing LRAT incubated with 2 mM ROH. Scale bars indicate 100 nm. ER is indicated by yellow lines in right panels; LDs are indicated by red lines in right panels. **(F)** Widefield microscopy of Erg6-mCherry expressing WT yeast cells expressing LRAT. Two time series of UV autofluorescence (white), Erg6-mCherry (red), and brightfield taken 2, 4, 6, and 8 min after addition of 2 mM ROH. Arrows indicate LDs that become UV^+^ over time. Scale bars indicate 5 µm.

TAG synthesis is a driving force in LD biogenesis and requires the transfer of activated fatty acids to DAG (Kassan et al., 2013). Acyl–coenzyme A (CoA), however, is not involved in the transesterification reaction of LRAT. To determine whether LRAT requires TAG synthesis for formation of RE-containing LDs, we incubated cells with triacsin C, which inhibits acyl-CoA synthesizing activities (Igal et al., 1997). In the presence of triacsin C, the number of LDs was strongly reduced in both the presence and absence of OA (Fig. 2 A). In the presence of ROH, however, formation of large LDs was still observed.

**Figure S2.**
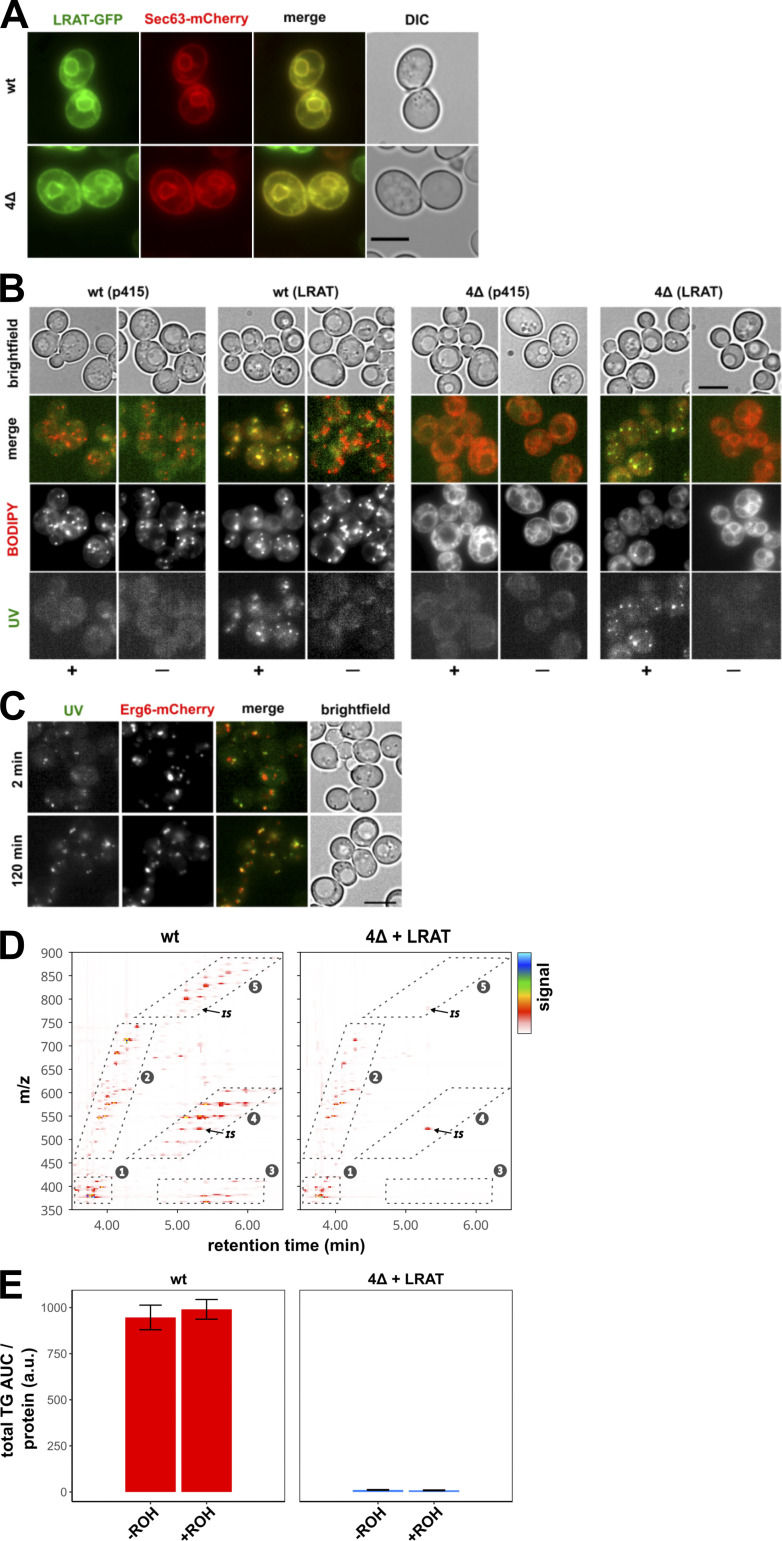
**LD formation in the absence of preexisting LDs. (A)** Widefield microscopy of WT and 4Δ yeast cells expressing LRAT-GFP (green) and Sec63-mCherry (red), showing the differential interference contrast (DIC; white) and fluorescence channels (colors and merge). **(B)** Widefield microscopy of WT and Δ4 yeast cells, with or without expressing LRAT, 2 h after incubation with or without 2 mM ROH (− or +). After staining, images of UV autofluorescence (green), BODIPY (red), and brightfield were taken. **(C)** Widefield microscopy of WT yeast cells expressing LRAT and Erg6-mCherry (red). Cells were imaged 2 (top) or 120 (bottom) min after addition of 2 mM ROH. Images of UV autofluorescence (green), BODIPY (red), and brightfield (white) were taken. Scale bars indicate 5 µm. **(D)** LC-MS contour plots of retention times between 3.5 and 6.5 min (x axis) and m/z values between 350 and 900 (y axis). Detector signal is represented by color code (see key at right). Numbered boxes indicate areas with ions from (1) sterols, (2) DAGs and ceramides, (3) SEs and TAGs [M+H-2RCOOH]^+^, (4) TAG [M+H-RCOOH]^+^, and (5) TAG [M+H]^+^. IS, internal standard TAG (15:0/15:0/15:0). Representative samples of WT (left) and 4Δ+LRAT (right) yeast strains, incubated with 2 mM ROH, are shown. (For experimental details, see Fig. 2). **(E)** Relative quantification of summed peak area of all TAG ions except internal standard from box 5 (D) of WT (left panel; red) and Δ4+LRAT (right panel; marine blue) yeast cells in the presence or absence of 2 mM ROH. AUC, area under the curve. Bar plot indicates mean ± SD.

Some LDs could still be observed in cells treated with triacsin C, however, and therefore we also made use of another model system, a *Saccharomyces cerevisiae* mutant strain that lacks the four enzymes responsible for producing TAG: Lro1, Dga1, steryl ester (SE), and acyl transferase–related enzymes 1 and 2 (Are1 and Are2, respectively). This mutant strain does not contain LDs (Sandager et al., 2002). After introduction of human LRAT into *lro1Δ dga1Δ are1Δ are2Δ* cells (hereafter 4Δ cells), LRAT-GFP colocalizes with Sec63-mCherry, an ER marker (Fig. S2 A). The resulting LRAT-expressing yeast cells (4Δ LRAT) were able to synthesize REs after the addition of ROH (Fig. 2 B) and were devoid of LD structures in the absence of exogenous ROH (Figs. 2 C and S2 B). Upon addition of ROH to 4Δ LRAT yeast cells, we observed autofluorescent LD-like structures that colocalized with the LD markers Erg6 (Figs. 2 C and S2 C) and BODIPY (Fig. S2 B). EM examination of the LDs generated in 4Δ LRAT yeast cells revealed the presence of bona fide LDs with a cytosolic orientation that were morphologically indistinguishable from TAG/SE-filled LDs generated in WT yeast cells (Fig. 2, D and E). These results demonstrate that LRAT induces the formation of LDs in the absence of other LDs or TAGs (Fig. S2, D and E). To determine whether REs can also partition into existing LDs, we determined the colocalization of RE-positive LDs (UV^+^) with all LDs (Erg6^+^) immediately after addition of ROH to WT yeast cells containing TAG-filled LDs and expressing LRAT. As shown in Fig. 2 F, 2 min after addition of ROH, colocalization of Erg6^+^ LDs with UV^+^ LDs was observed. In addition, UV^─^ LDs become UV^+^ in the next 6 min (indicated with arrows), indicating that REs are also transferred into existing LDs.

### Spontaneous nucleation and lens formation of REs in lipid bilayers

**Figure 3.**
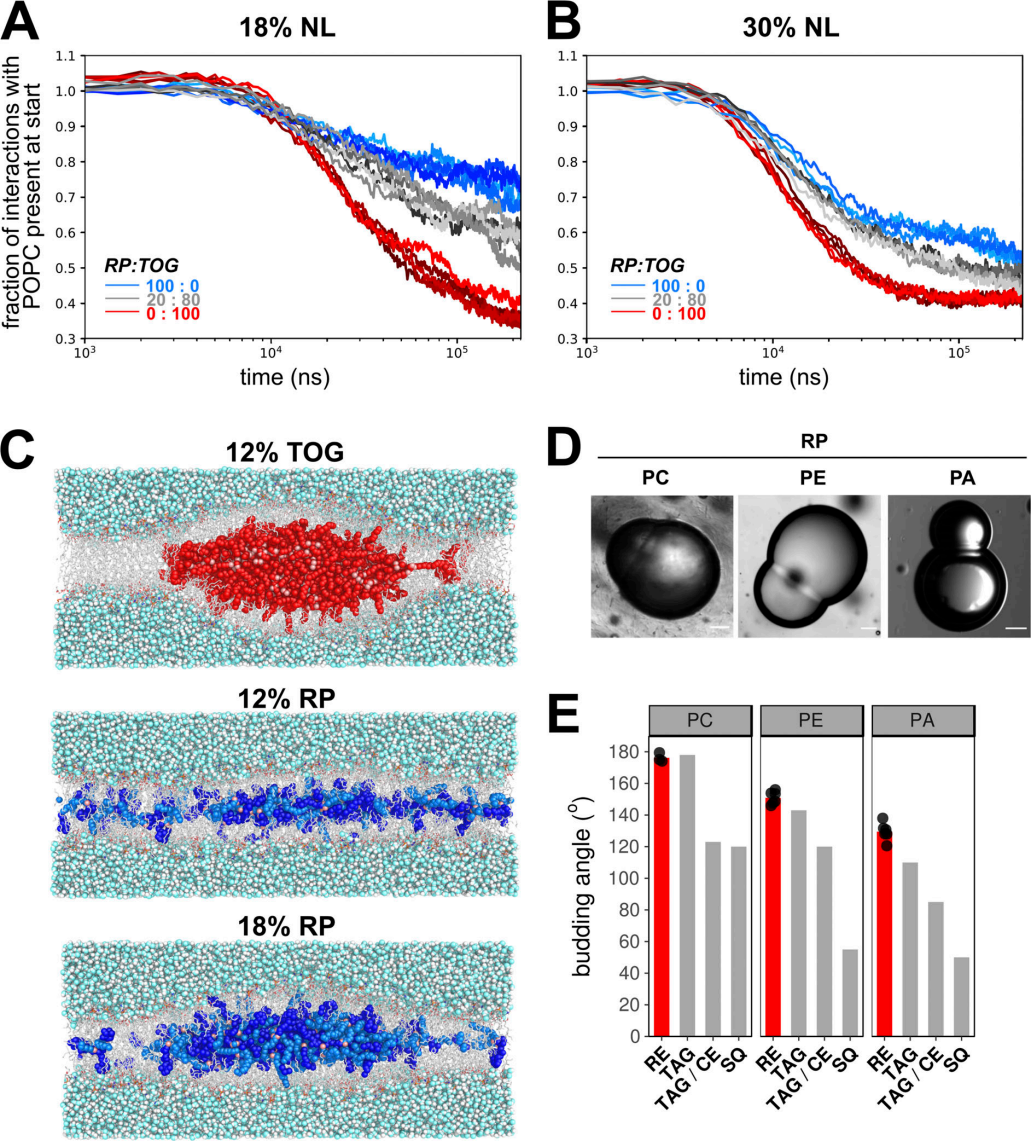
**Nucleation and budding properties of REs.** **(A and B)** Coarse-grained MD simulations of lens formation by 150 (18%; A) and 250 (30%; B) neutral lipids (NL) per leaflet in a POPC membrane. Colors indicate the neutral lipid composition with marine blue for pure RE and red for pure TOG. The progress of lens formation is shown as fractional loss of interactions between the neutral lipids and POPC as a function of time (logarithmic scale). **(C)** Snapshots of systems with 12% TOG (top), 12% RP (middle), and 18% RP (bottom) obtained after backmapping and equilibrating the corresponding CG systems to charmm36 all-atom models. TOG carbon atoms are colored red, and oxygen atoms are colored pink. For RP, the retinyl moiety is colored bright blue, the palmitoyl group is colored marine blue, and the oxygen atoms are colored pink. **(D)** Brightfield microscopy images of DIBs of droplets containing neutral lipid RP and lipid surfactants PC, PE, or PA. Scale bars indicate 20 µm. **(E)** Comparison of quantified budding angles (mean ± SD) of RP-containing droplets with lipid surfactant PC (176.2 ± 2.8), PE (150.9 ± 4.0), or PA (129.5 ± 5.7) versus reported budding angles of TAG- and squalene (SQ)-containing droplets (Ben M’barek et al., 2017). Bar plot represents mean of at least eight individual measurements, which are shown as black dots.

The formation of lenses in TAG-containing membranes has been studied in molecular dynamics (MD) simulations (Khandelia et al., 2010; Ben M’barek et al., 2017). Here we built upon that approach and used multiscale (CG/AA) MD simulations of 1-palmitoyl-2-oleoyl-*sn*-glycero-3-phosphocholine (POPC) membranes with different amounts of trioleoylglycerol (TOG) and retinyl palmitate (RP) to assess the propensity of lens formation in those systems. In CG-MD setups containing only POPC and TOG, lens formation was consistently observed and completed within 100 ns of simulation. In contrast, setups with only RP in POPC took considerably longer to nucleate (Fig. 3 A) and more often failed to form lenses on the time scale used for the simulations (250 ns). Lenses formed by RP were also typically less well defined, with more RP remaining dispersed throughout the membrane. Mixtures of TOG and RP showed an intermediate efficiency of lens formation (Fig. 3, A and B). Cross-sections of AA-MD simulations of POPC membranes with TAG or RP confirmed these results (Fig. 3 C). These simulations imply that RP has a lower propensity to self-aggregate and hence has a higher nucleation barrier than TOG. Analysis of intermolecular contacts between POPC, RE, and TOG suggests that the smaller number of ester groups relative to the aliphatic chains contributes to this lower propensity, because they were found to cluster toward the middle of the membrane (Fig. 3 C) for both TOG and RE molecules.

Methods to directly measure the nucleation barrier do not exist, but in a setting with the same phospholipid composition, the monolayer surface tension will be a driving force that affects nucleation (Ben M’barek et al., 2017; Deslandes et al., 2017; Thiam and Forêt, 2016). We measured the tension of artificial LDs containing either RP or TOG, and tensions were considerably lower in LDs made of RP than in their TOG LD counterparts (Table 1). Droplets surrounded by dioleoylphosphatidylcholine (DOPC) with maximum phospholipid packing showed the same trend. These measurements suggest that RP LDs have higher nucleation barriers, in agreement with the results obtained by CG-MD.

**Table 1.** Interfacial tension values by drop tensiometry

System	Tension (mN/m)
TOG/H_2_O	32
RP^a^/H_2_O	8–14
TOG/DOPC/H_2_O	0.5–0.6
RP^a^/DOPC/H_2_O	0.08–0.2

a Based on density provided by manufacturer, 0.90–0.95 g/ml.

The efficiency of the subsequent budding process of RE-containing LDs can be studied by determination of budding angles in droplet-embedded vesicles or droplet interface bilayers (DIBs) containing neutral lipids (Chorlay and Thiam, 2018; Ben M’barek et al., 2017). We compared the budding angles of the RP-containing lipid phase with reported values of TAG-filled LDs (Fig. 3, D and E). The budding angles of RP^+^ droplets with PC and phosphatidylethanolamine (PE) monolayers did not differ from reported values of their TOG^+^ counterparts (Fig. 3 E); angles in the case of phosphatidic acid (PA) were only slightly higher. These data suggest that, for the budding process itself, no difference is observed between RP-filled LDs and TAG-filled LDs.

### Seipin does not affect the size of RE-filled LDs

**Figure S3.**
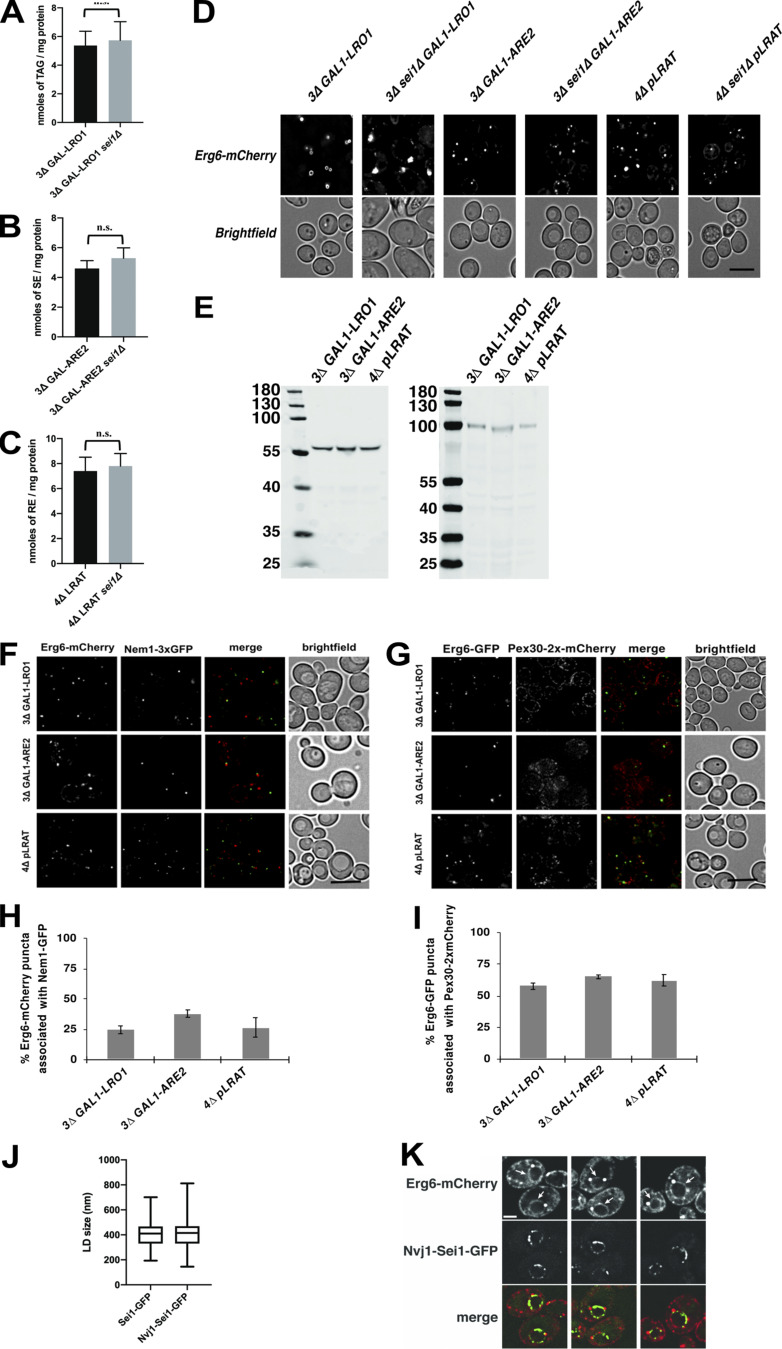
**Neutral lipids in cells with or without seipin. (A–C)** Bar graphs showing TAG (A), SE (B), or RE (C) levels after induction with galactose (A and B) or ROH (C). Neutral lipids were measured with LC coupled to an ELSD. Cells were grown as described for Fig. 4 A; the amounts of neutral lipid indicate mean ± SD. Statistical significance was determined by Welch’s *t* test. ***, P < 0.001. **(D)** Fluorescence microscopy images of single neutral lipid strains with or without seipin expressing the LD marker Erg6-mCherry. Strains containing genes under the *GAL1* promoter were grown in medium containing raffinose and galactose for 24 h and visualized live. Strains containing the plasmid expressing LRAT were grown in SC medium with ROH for 24 h. Single deconvolved images are shown from Z-stacks of 20 images. Scale bars indicate 5 µm. **(E)** The indicated strains containing plasmids that express Sei1-GFP or Nvj1-Sei1-GFP were grown as described in Fig. 5, A and B. Extracts were separated by SDS-PAGE and immunoblotted with anti-GFP antibodies. The expected molecular weights of the Sei1-GFP and Nvj1-Sei1-GFP fusions are 60 kD and 99 kD, respectively. Numbers are sizes of the molecular weight markers. **(F–I)** Association of nascent LDs with Nem1 (F and H) or Pex30 (F and H). Strains were grown and imaged as in Fig. 4 A. Scale bars indicate 5 µm. Bar graphs in H and I show mean ± SD from 100 cells from 3 independent experiments for the results shown in F and G, respectively. **(J)** A 3Δ *sei1*Δ* GAL1-LRO1* strain expressing Erg6-mCherry and either Sei1-GFP or Nvj1-Sei1-GFP was grown in SC medium with raffinose; galactose was added to the medium; and, 4 h later, the cells were visualized by fluorescence microscopy. The size of 100 LDs (Erg6-mCherry signal) was determined from 3 independent experiments. There is no statistically significant difference between the samples using Student’s *t* test. Compare these results with *GAL1-LRO1* strains in Fig. 4 B. (**K)** 3Δ *GAL1-LRO1* cells expressing Erg6-mCherry and Nvj1-Sei1-GFP were grown at 30°C in SC medium with raffinose; galactose was added the medium; and, 4 h later, the cells were visualized. Erg6-mCherry is on both the ER and the surface of LDs. The nucleus is indicated with an arrow. Three examples are shown. Scale bar indicates 1 µm.

**Figure 4.**
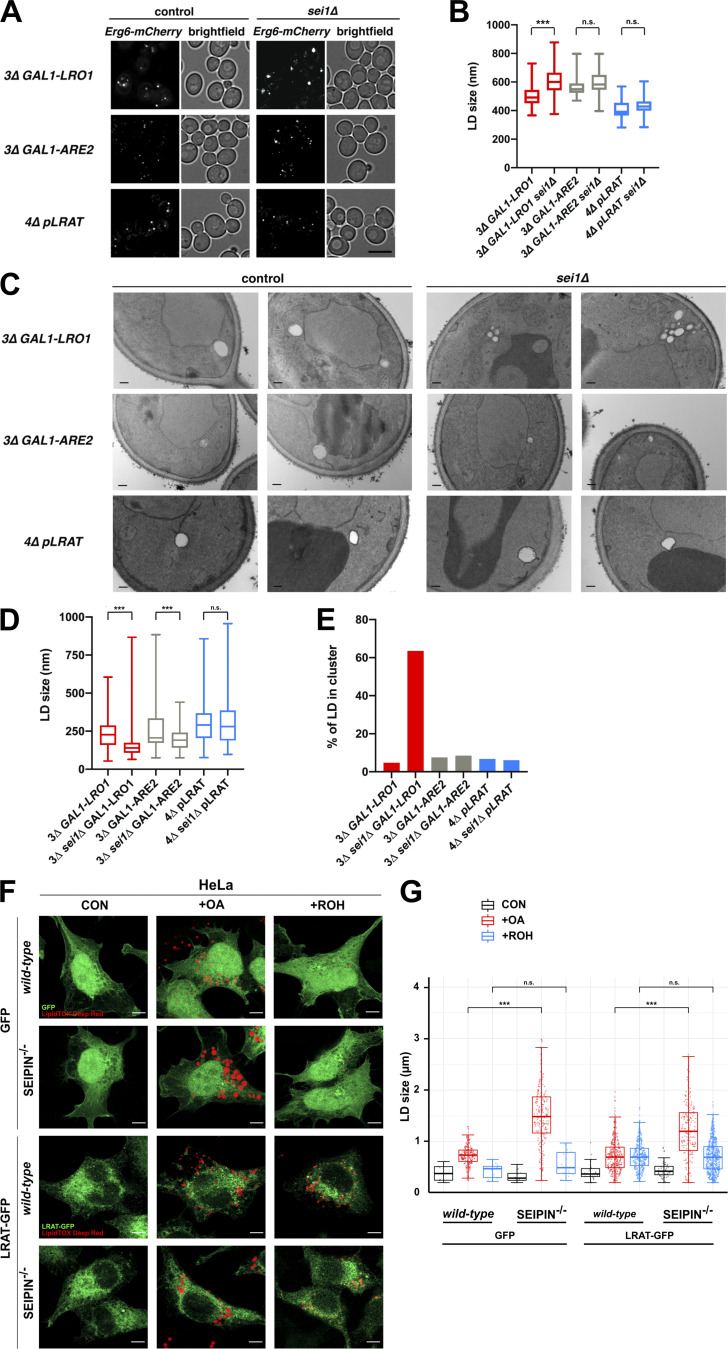
**Seipin deletion does not alter the size or clustering of RE-filled LDs.** **(A)** Fluorescence microscopy images of yeast strains with (control) or without (*seiΔ1*) seipin and expressing the LD marker Erg6-mCherry. Cells were grown in SC medium with raffinose. TAG or SE production was induced by adding galactose to the 3Δ *GAL1-LRO1* and 3Δ *GAL1-ARE2* cells. These strains were imaged 4 and 6 h after galactose addition, respectively. 4Δ pLRAT cells were grown in SC medium and imaged 2 h after the addition of ROH. Full-projection images are shown from Z-stacks of 20 deconvolved images. Scale bars indicate 5 µm. **(B)** Quantification of LD diameters from the conditions in A (at least 100 LDs per condition from three independent experiments). Statistical significance was determined by Student’s *t* test. **(C)** EM images of the same conditions as in A. Scale bars indicate 200 nm. **(D)** Box plots with quantified LD diameters from the conditions in C (number of LDs counted: 3D *GAL1-LRO1*, *n* = 213; 3Δ *sei1*Δ *GAL1-LRO1*, *n* = 358; 3Δ *GAL1-ARE2*, *n* = 219; 3Δ *sei1*Δ *GAL1-ARE2*, *n* = 220; 4Δ pLRAT, *n* = 185; 3Δ *sei1*Δ pLRAT, *n* = 203). Statistical significance was determined by Mann-Whitney tests of two independent experiments. **(E)** Percentage of LDs present in clusters containing three or more LDs, based on the LD quantification shown in D.** (F)** Fluorescence microscopy images of WT and seipin^−/−^ HeLa cells transiently expressing GFP (top) or LRAT-GFP (bottom, both shown in green), incubated with medium control, 100 µM OA, or 10 µM ROH. LDs are visualized in red (LipidTOX Deep Red). Scale bars indicate 10 µm. **(G)** Quantification of LD diameters from the conditions in F (number of LDs counted in the respective conditions: *n* = 6, 220, 12, 14, 213, 16, 24, 351, 387, 54, 170, 627). CON, control; +OA, +oleic acid; +ROH, +retinol. Statistical significance was determined by Mann-Whitney test of one representative experiment. ***, P < 0.001.

In yeast, cells lacking seipin have clusters of small LDs or supersized LDs (Fei et al., 2008; Szymanski et al., 2007). To determine the involvement of seipin in the assembly of RE-containing LDs, we compared diameters of newly formed LDs composed of only RE, TAG, or SE in cells that contain or lack seipin (*sei1*Δ). Cells that express or lack seipin produced about the same amount of neutral lipid during the induction period (Fig. S3, A–C). As expected, in cells that produce exclusively TAG-filled LDs (3Δ *GAL1-LRO1*), deletion of seipin resulted in abnormal LDs (Fig. 4 A, upper panel; and Fig. 4 B). In contrast, the mean size of RE-filled LDs (4Δ pLRAT) was not significantly altered by deletion of seipin (Fig. 4 A, bottom panels; and Fig. 4 B). Also, the size of LDs composed of SE (3Δ *GAL1-ARE2*) was not significantly different (Fig. 4 A, middle panels; and Fig. 4 B). Even when these strains were grown for 24 h, LDs composed of either SE or RE were not abnormal in *sei1*Δ cells (Fig. S3 D). EM analysis revealed clusters of small LDs in 3Δ *GAL1-LRO1 sei1Δ* cells (Fig. 4 C, top panels; and Fig. 4, D and E), suggesting that the seemingly abnormally large LDs observed by fluorescence microscopy were artifacts caused by the diffraction limit of light microscopy. Eliminating seipin had no effect on the size of RE-LDs (Fig. 4 C, bottom panels; and Fig. 4, D and E), whereas it caused a small but significant decrease in the size of LDs composed of SE (Fig. 4 C, middle panels; and Fig. 4, D and E). The seipin-independent assembly of RE-containing LDs was confirmed in a mammalian system by expressing LRAT-GFP in HeLa cells lacking seipin (Yan et al., 2018). As expected, LD formation with OA resulted in enlarged LDs in the absence of seipin (Fig. 4, F and G). Expression of LRAT-GFP in HeLa cells allowed the induction of LD formation with ROH, and, under these conditions, the presence or absence of seipin had no effect on LD size (Fig. 4, F and G). Taken together, these findings indicate that seipin does not affect the biogenesis of LDs composed of RE.

### Biogenesis of LDs without TAG is independent of seipin

**Figure 5.**
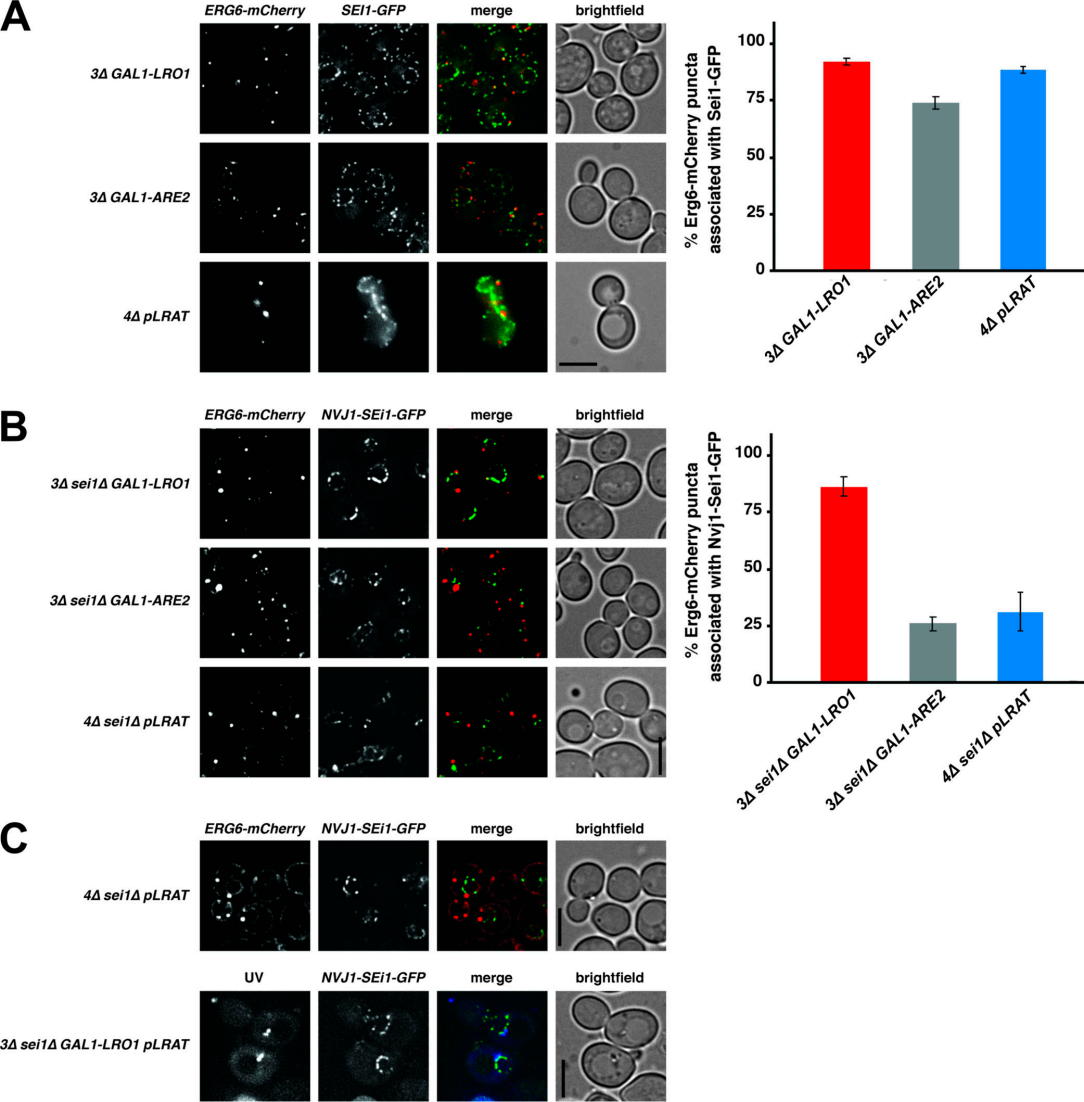
**Seipin does not determine the biogenesis site of LDs filled with RE or SE.** **(A–C)** Fluorescence microscopy images of yeast strains expressing Erg6-mCherry (left panels) and Sei1-GFP (A, middle panels) or Nvj1-Sei1-GFP (B and C, middle panels). **(A and B)** Cells were grown as described in Fig. 4 A to induce the production of TAG, SE, or RE. Bar graphs (A and B) show the percentage of LDs (Erg6-mCherry puncta) associated with Sei1-GFP (A) or Nvj1-Sei1-GFP (B) foci. Graphs show mean ± SD from 100 cells (3 independent experiments). **(C)** Cells were grown in SC medium with raffinose. After the addition of galactose and 60-min incubation, ROH was added, and cells were grown for an additional 10 min and imaged. RE-containing LDs were visualized by UV autofluorescence. All microscopy images are full projections of single, deconvolved images from 20-image Z-stacks. Scale bars indicate 5 µm.

Seipin forms foci in the ER that often associate with nascent LDs (Wang et al., 2016), and relocalization of seipin to other ER sites causes LD biogenesis to occur there (Chung et al., 2019; Salo et al., 2019). We quantified the number of LDs that were associated with seipin-GFP foci in yeast cells producing LDs containing newly produced TAG, RE, or SE. The three different yeast strains expressed similar levels of seipin-GFP (Fig. S3 E), and all three LD types showed a similar seipin association of ∼85% (Fig. 5 A). The LDs also associated with two other markers of LD biogenesis sites, Nem1 and Pex30, to a similar degree (Fig. S3, F–I). While yeast TAG-synthesizing enzymes become enriched at LD biogenesis sites when LD biogenesis is induced (Choudhary et al., 2020), LRAT does not (Fig. S2 A), suggesting that seipin has affinity for nascent LDs in the ER, regardless of which neutral lipid is present.

Because seipin determines the biogenesis sites of LDs in mammalian cells (Chung et al., 2019; Salo et al., 2019), we next determined whether restricting seipin to a subcompartment of the ER affects the biogenesis site of RE-filled LDs. Nucleus–vacuole junction protein 1 (Nvj1) is an ER membrane protein normally present at contact sites between the outer nuclear membrane and the vacuole (Pan et al., 2000). We fused Sei1-GFP to the first 260 amino acids of Nvj1 and expressed the construct in strains lacking endogenous seipin. Expression of Nvj1-Sei1-GFP resulted in functional complementation of the seipin-knockout (KO) phenotype (Fig. S3 J versus Fig. 4 B) and resulted in seipin localization in the perinuclear ER (Fig. 5 B and Fig. S3 K). Most newly formed TAG-filled LDs are associated with Nvj1-Sei1-GFP (Fig. 5 B), indicating that seipin determines the biogenesis site of this type of LD. Strikingly, most newly formed LDs composed of either RE or SE were not associated with Nvj1-Sei1-GFP (Fig. 5 B). These findings suggest that LDs composed of RE or SE form independently of seipin, even when this protein is present in cells.

To test whether seipin can affect the distribution of REs in cells with preexisting TAG^+^ LDs, we expressed LRAT in 3Δ *GAL1-LRO1* cells that also expressed Nvj1-Sei1-GFP (instead of endogenous seipin) and the LD marker Erg6-mCherry. First, we added galactose to induce TAG production and incubated the cells for 60 min. Next, we added ROH to initiate RE production and examined the cells after 10 min. Under these conditions, we did observe UV^+^ LDs localized to the perinuclear ER (Fig. 5 C). Our combined findings show that RE^+^ LDs can be formed independently of seipin, but seipin may indirectly affect the distribution of RE by affecting the partitioning of RE into preexisting, seipin-mediated TAG^+^ LDs.

### Conclusions

Our findings show the existence of a seipin-independent route for LD biogenesis driven by production of the neutral lipid RE by LRAT. The idea that seipin does not participate in the biogenesis of all LDs is consistent with a recent study showing that LDs form independently of seipin in the inner nuclear membrane (Sołtysik et al., 2021). We propose that formation of RE-filled LDs is driven in part by RE itself. This is consistent with our MD simulations and in vitro experiments demonstrating that RE spontaneously nucleates in membrane bilayers to form nascent LDs, albeit less efficiently than TAG, and by the finding that yeast cells lacking the machinery to synthesize TAG and SE nonetheless produce RE-filled LDs. We propose that RE also drives LD formation in HSCs. The expression of LRAT in quiescent HSCs coincides with the presence of large RE-containing LDs. When LRAT expression is reduced (in activated HSCs; Fig. 1 E) or absent in LRAT^−/−^ mice (Ajat et al., 2017; O’Byrne et al., 2005), HSCs lack their characteristic UV^+^ (i.e., RE-containing) LDs. A direct demonstration that seipin does not play a role in formation of RE-containing LDs in HSCs is not possible, because seipin-KO mice develop a progressive steatotic phenotype early after birth, which makes it impossible to assess LD size in HSCs in these mice (Prieur et al., 2013).

Our data suggest that the role of seipin in LD formation is determined in part by the neutral lipids being stored in LDs. Seipin enhances TAG sequestering inside ringlike oligomeric structures (Chung et al., 2019; Salo et al., 2019; Prasanna et al., 2021; Zoni et al., 2021). Seipin oligomers can trap TAGs in the ER bilayer via luminal hydrophobic helices and promote TAG clustering at low concentrations (Prasanna et al., 2021). It remains to be established whether a similar mechanism exists for the efficient sequestering of REs in the ER membrane before the formation of RE-filled LDs. The presence of RE-rich and RE-deficient LDs within the same cell may suggest a similar phenomenon for trapping REs. In this respect, the reason for seipin association with RE-filled LDs is not clear. We showed that seipin does affect the transfer of REs into preexisting TAG-filled LDs. By forming oligomeric complexes, seipin could bring different types of associated LDs in close proximity. We speculate that neutral lipid-specific molecular machineries may exist that specifically catalyze and regulate the biogenesis of subtypes of LDs.

## Materials and methods

### HSC isolation and cell culturing

HSCs were isolated from 10–12-wk-old male mice (C57BL/6J background; WT pups from crossed LRAT^+/−^ heterozygote mice; Liu and Gudas, 2005) as described before (Riccalton-Banks et al., 2003). Animals were handled according to governmental and international animal experimentation guidelines and laws. Experiments were approved by the animal experimentation committee (Dierexperimentencommissie) of Utrecht University (2013.III.02.016). After isolation, cells were protected from light and cultured on coverslips in 24-well plates (Nunc) in DMEM supplemented with 10% FBS, 100 U/ml penicillin, and 100 µg/ml streptomycin (all obtained from Gibco/Invitrogen).

CHO-k1 cells were cultured in Ham’s F-12 medium supplemented with 7.5% FBS, 100 U/ml penicillin, and 100 µg/ml streptomycin. The human HSC line LX-2 cells (kindly donated by Dr. Friedman, Icahn School of Medicine at Mount Sinai, New York, NY) were grown in DMEM containing 10% FBS, 100 U/ml penicillin, and 100 µg/ml streptomycin. All cells were maintained in a humidified incubator (5% CO_2_) at 37°C. Depending on the experiment, cells were incubated with ROH (MilliporeSigma; stocks of 30 mM in EtOH) and OA (MilliporeSigma) coupled to fatty acid–free BSA (MilliporeSigma; stocks of 10 mM fatty acid in 12% BSA) and/or triacsin C (Cayman; stocks of 1 mg/ml in DMSO).

WT and seipin-KO HeLa cells (Yan et al., 2018) were seeded onto a six-well plate laid with a coverslip in DMEM supplemented with 10% FBS and 1% penicillin/streptomycin. Cells were then transfected with LRAT-GFP or GFP vector using Lipofectamine LTX reagent (Thermo Fisher Scientific). 8 h after transfection, cells were lipid starved in DMEM with 1% lipoprotein-deficient serum containing inhibitors of DGAT1 (5 µM; PF-04620110), DGAT2 (5 µM; PF-06424439), and acyl-CoA cholesterol acyltransferase (4.2 µM, Sandoz 58-035; all obtained from MilliporeSigma) for 24 h. After starvation, cells were washed in PBS three times before incubation in 1% lipoprotein-deficient serum media supplemented with 100 µM BSA-coupled OA or 10 µM ROH for 16 h. Cells were washed in PBS twice, followed by fixation with 4% PFA (Electron Microscopy Sciences) for 15 min. After fixation, cells were washed three times with PBS, and LDs were then stained with HCS LipidTOX Deep Red Neutral Lipid Stain (Thermo Fisher Scientific) in PBS for 1 h. Images were taken on a Zeiss 900 confocal microscope using a 40× oil immersion objective lens.

### ROH handling

ROH (MilliporeSigma) was dissolved in ethanol (30 mM) and stored in 50-µl aliquots at −80°C. We routinely measured UV absorption spectra of ROH stocks before use and calculated the current stock concentration by making use of the molar extinction coefficient of ROH in EtOH (52,480 mol L^−1^ cm^−1^ at 325 nm; Ross, 1981). In addition, we made use of the observation that after exposure to UV light, a peak emerged at ∼240 nm, accompanied with a decrease at ∼325 nm. We routinely monitored E_325_/E_240_ ratios and used exclusively stocks with a ratio of 5 or higher, which was estimated to correspond to ROH integrity of ∼70%.

### Generation of stable cell lines

Human LRAT cDNA was synthesized and cloned into a pcDNA3.1(+) vector (Clontech) by a third party (GeneArt/Thermo Fisher Scientific). In addition, the LRAT sequence was cloned into a pEGFP-N2 vector (Clontech), resulting in LRAT fused to the N-terminus of GFP. CHO-k1 and LX-2 cells were transiently transfected by Lipofectamine 2000 reagent (Thermo Fisher Scientific) according to the manufacturer’s instructions. Stable CHO-k1 cells expressing the various LRAT variants or control GFP were generated as follows. Cells (2 × 10^6^) were electroporated in PBS with 15 µg DNA and a pulse of 260 V. After plating, cells were cultured for 48 h in the absence and for 3 wk in the presence of 1 mg/ml G-418 selection antibiotic (Thermo Fisher Scientific). Stable cells expressing GFP fusion proteins were trypsinized, and single GFP^+^ cells with forward and side scatter properties similar to those of their nontransfected counterparts were plated into 96-well plates by FACS (Influx Cell Sorter; BD Biosciences). A GFP-negative gate was chosen to select monoclonal stable cells expressing nonfluorescent proteins. After plating, the monoclonal cells were allowed to grow until wells reached confluency. Clones with comparable morphology to that of parental CHO-k1 cells (cell size, nucleus, LDs) were selected. The absence or presence of LRAT enzymatic activity in LRAT-, GFP-, and LRAT-GFP–expressing clones was confirmed by determination of REs (see below) and UV^+^ autofluorescent LDs in combination with GFP fluorescence by FACS (FACSCanto II; BD Biosciences) after incubation of cells with ROH.

### Confocal microscopy of mammalian cells

Cells were plated on Lab-Tek II eight-chamber slides (Thermo Fisher Scientific) and incubated as described in the figure legends. Cells were fixed in 4% PFA (Electron Microscopy Sciences). Subsequently, cells were stained with either DAPI, BODIPY 493/503 (Thermo Fisher Scientific), and/or LD540 (kindly donated by Dr. C. Thiele, Biochemistry and Cell Biology of Lipids, LIMES, University of Bonn, Bonn, Germany; Spandl et al., 2009). Staining for immunofluorescence was performed with anti-desmin or anti–α-smooth muscle actin (both from Thermo Fisher Scientific), followed by goat anti-mouse Alexa Fluor 647 or donkey anti-rabbit Alexa Fluor 647 (Life Technologies). Cells were mounted with FluorSave reagent (Calbiochem) and subsequently imaged with a Leica TCS SPE laser scanning spectral confocal microscope or a Nikon A1R confocal microscope using preset settings for the representative dyes. For the detection of retinoid autofluorescence, presets for DAPI were used.

### 3D-SIM

After treatment, cells were stained with DAPI and HCS LipidTOX Red Neutral Lipid Stain (Thermo Fisher Scientific) and mounted with VECTASHIELD Antifade Mounting Medium (Vector Laboratories). 3D-SIM was performed using a Deltavision OMX-V4 Blaze (GE Healthcare) setup equipped with four scientific complementary metal–oxide–semiconductor cameras (PCO). Immersion oil with a 1.516 refractive index (GE Healthcare) was placed on the 60× objective (Olympus U-PLAN APO; NA 1.42). Fluorophores were excited with a 405-nm diode (Vortran Stradus; 100 mW) and a 568-nm optically pumped semiconductor laser (Coherent; 100 mW) modulated to 1% by neutral density filters. System-supplied filter blocks were used to acquire fluorescence of DAPI (excitation, 382–409 nm; emission, 421–450 nm) and LipidTOX Red (excitation, 561–580 nm; emission, 591–627 nm). Raw images were processed using softWoRx software (Applied Precision) with system optical transfer functions predetermined with 100-nm fluorescent polystyrene beads (Thermo Fisher Scientific) and camera alignment parameters for the different channels. Acquired images were deconvolved using default settings (omitting Wiener filtering and background subtraction), including negative values, and intensities were linearly adjusted. Images in the figures are supplied as maximum-intensity projections.

### Growth and fluorescence microscopy of yeast

Yeast strains and plasmids used in this study are described in Table S1. Yeasts were grown at 30°C in synthetic complete (SC) media containing 0.67% yeast nitrogen base without amino acids (United States Biological), 2% glucose, and an amino acid mix (United States Biological). Where indicated, 2% raffinose and 2% galactose were used in place of glucose. ROH (MilliporeSigma) was added to the medium at 4 mM together with 1% IGEPAL CA-630 (MilliporeSigma). Cells were stained with 0.5 µg/ml BODIPY 493/503 (Invitrogen) for 10 min and washed once with PBS. Tunicamycin (MilliporeSigma) was added to media at a final concentration of 0.4 µg/ml from a stock of 5 mg/ml in DMSO. Yeasts were imaged at 30°C in an environmental chamber with a DeltaVision Spectris (Applied Precision) comprising a widefield inverted epifluorescence microscope (IX70; Olympus), a 100 Å/NA 1.4 oil immersion objective (UPlanSAPO; Olympus), and a charge-coupled device CoolSNAP HQ camera (Photometrics). Where indicated, images were deconvolved using the conserved ratio method and softWoRx (Applied Precision).

### Retinoids and neutral lipid determination by liquid chromatography/tandem mass spectrometry (LC-MS/MS) or LC with an evaporative light-scattering detector (ELSD)

Lipids were extracted as previously described (Bligh and Dyer, 1959). To avoid photoisomerization and oxidation of the retinoids, extractions were performed under red light and in amber tubes. In addition, 1 nmol butylated hydroxytoluene was added to every sample. As an internal standard, 250 pmol retinyl acetate in MeOH/CHCl_3_ (1:1 vol/vol) was added. The combined chloroform phases were dried under nitrogen and stored at −20°C until further analysis.

Extracts were dissolved in MeOH/CHCl3 (1:1) and stored in amber autosampler vials. To measure retinoids, samples were injected and separated on a 250 × 3.0–mm Synergi 4-µm Max-RP 80A column (4-µm particle size; Phenomenex) with a flow rate of 350 µl min^−1^. To this end, a gradient (solvent A, acetonitrile:water [95:5]; solvent B, acetone:chloroform [85:15]; 0 min, 90% A; 5 min, 40% A; 17 min, 0% A; 19 min, 90% A; 25 min, 90% A) was generated by a Flexar ultra-HPLC system (PerkinElmer). The column outlet was connected to a triple-quadrupole MS system (SCIEX API 4000 QTRAP; MDS Analytical Technologies/Applied Biosystems) with an atmospheric pressure chemical ionization source (set to 500°C). Multiple reaction monitoring in positive ion mode was used to detect RE species with settings and mass-to-charge (m/z) transitions as described before (Ajat et al., 2017). Chromatographic peaks were integrated and quantified using Analyst software version 1.4.3 (Applied Biosystems).

To measure other neutral lipids (sterols, TAGs, SEs), samples were injected and separated on a Kinetex/HALO C8 column (2.6 µm, 150 × 3.00 mm; Phenomenex). A gradient of methanol/H_2_O (5:5 vol/vol; solvent A) and methanol/isopropanol (8:2 vol/vol; solvent B) was generated by an Infinity II 1290 ultra-HPLC system (Agilent Technologies) and with a constant flow rate of 600 µl min^−1^ (0 min, 100% A; 2 min, 0% A; 8 min, 0% A; 8.5 min, 100% A; 10 min, 100% A). Lipids were measured using atmospheric pressure chemical ionization in positive mode coupled to an Orbitrap Fusion mass spectrometer (Thermo Fisher Scientific). Vendor data files were converted to mzML format with msConvert (part of ProteoWizard version 3.0.913) and processed with XCMS Online version 3.7.0 (Tautenhahn et al., 2012).

For the neutral lipid quantification, as shown in Fig. S3, A–C, lipids were extracted as described previously (Parks et al., 1985) after lysing cells with glass beads in a Precellys 24 homogenizer (Bertin Instruments). Lipids were dried under N_2_, resuspended in hexane, and injected onto a Zorbax CN (Agilent Technologies) 150 × 4.6–mm (5-µm) column on a 1290 Infinity II LC system (Agilent Technologies). Mobile phase A was hexane, and mobile phase B was tert-butyl methyl ether, and the flow rate was 1 ml/min. Solvent B was set at 3% for 3 min, then increased to 20% by 12 min and 100% by 17 min, and held at 100% for 5 min. The column was allowed to reequilibrate with 3% solvent B for 5 min before the next injection. Lipids were detected with a 1290 Infinity II ELSD (Agilent Technologies) with an evaporator temperature of 90°C, nebulizer temperature of 50°C, and a gas (N_2_) flow rate of 2 liters/min. The peaks containing neutral lipids were identified by comparison with the retention times of known standards (MilliporeSigma). Calculation of absolute amounts was based on external standards.

### LRAT activity assay

LRAT activity in homogenates expressing LRAT-GFP was performed as described previously (Golczak et al., 2015). Briefly, cells were cultured overnight in T-75 culture flasks (CELLSTAR; Greiner Bio-One) under normal cell growth conditions. After being scraped in ice-cold PBS, cells were homogenized on ice with 26-gauge needles (BD Biosciences). Homogenates containing 200 µg total protein were mixed with reaction mix containing 5 mM DTT, 5 mM EDTA, 10 mM Tris-HCl (pH 8.0), 1% BSA, 0.2 µM ascorbic acid, 2 mM PC (7:0/7:0), and increasing amounts of ROH. Subsequently, mixtures were incubated for 60 min at 37°C in amber glass vials. Levels of retinyl heptanoate were determined by LC-MS/MS (see below).

### LRAT mRNA expression by quantitative PCR

Expression of LRAT mRNA was determined as described previously (Tuohetahuntila et al., 2017). Briefly, RNA was isolated with an RNeasy Micro Kit (Qiagen), and cDNA was synthesized with an iScript cDNA Synthesis Kit (Bio-Rad Laboratories). PCR amplifications were performed using a Bio-Rad detection system with iQ SYBR Green Supermix (Bio-Rad Laboratories). Gene expression was normalized against reference genes, and sequences of the primers are listed in Table S2.

### MD

MD simulations were performed using a sequential multiscale approach comprising Martini CG simulations for equilibration and subsequent conversion to charmm36 all-atom models using the *Backward* approach (Wassenaar et al., 2014). CG-MD simulations were set up and run using the docking assay for transmembrane components protocol (Wassenaar et al., 2015b), according to procedures for building membrane/solvent systems using *insane* (insert membrane; Wassenaar et al., 2015a) and for generating membrane/solvent/protein systems as described in Wassenaar et al. (2015b). Simulations for assessing the lens-forming propensity and speed were set up in a hexagonal prism unit cell with base length of 24 nm and height of 10 nm. Numbers or ratios of lipids were set as described in the legend of Fig. 3. All simulations were performed using the coarse-grained Martini 2.2 model (Marrink et al., 2004, 2007; Monticelli et al., 2008) and run in Gromacs 2018.x (Pronk et al., 2013) using the automated Martini workflow *martinate* (Wassenaar et al., 2013). The topology for the retinyl group was generated for Martini 2 on the basis of similarities with available ubiquinone/ubiquinol, β-carotene, and retinal models (provided by Dr. Paulo C. Telles de Souza, Molecular Microbiology and Structural Biochemistry, UMR 5086 CNRS and University of Lyon, Lyon, France). Simulations were run with different concentrations of RP and TOG for 250 ns. After 250 ns, the final frames were converted to charmm36 and shortly equilibrated to check the integrity and consistency of the models. After backmapping, the systems comprised 480,000–500,000 atoms.

### EM

For the EM shown in Fig. 2, yeast cells were grown in synthetic dropout media lacking leucine at 30°C to an OD_600_ of ∼0.6. IGEPAL (MilliporeSigma) was added to the cell cultures to a final concentration of 1% before addition of ROH (MilliporeSigma) to a final concentration of 2 mM. Cells were then incubated for 10 min at 30°C before being processed for EM as follows. Cells were chemically fixed, embedded with 12% gelatin, cryosectioned, and stained as previously described (Griffith et al., 2008). Sections were imaged in an FEI CM100bio electron microscope at 80 kV equipped with a digital camera (Morada; Olympus). Two different grids with sections obtained from the same preparation were statistically evaluated by counting 75 randomly selected cell profiles before determining the average number of LDs per cell section plus the SD between the two grids.

The EM for Fig. 4 was performed by the University of Texas Southwestern Electron Microscopy Core Facility. Strains were grown to an OD_600_ of 1 at 30°C, and 20 ml of cultures was mixed with an equal volume of 2× prefix solution at 30°C (4% glutaraldehyde in 0.2 M Pipes, 0.2 M sorbitol, 2 mM MgCl_2_, 2 mM CaCl_2_). After 5 min at RT, the cells were pelleted (1,000 ×*g* for 5 min), resuspended in 1× prefix solution, and shipped immediately at 4°C to the EM facility. The cells were processed as described previously (Wright, 2000) with some modifications. In brief, the cells were fixed in potassium permanganate, dehydrated, stained in uranyl acetate, and embedded in Spurr resin that was polymerized at 60°C overnight. The blocks were sectioned at 70 nm on a Leica Ultracut UCT 6 ultramicrotome (Leica Microsystems). Sections were post-stained with 2% uranyl acetate in water and lead citrate. Images were acquired on a Tecnai G2 Spirit TEM (FEI) equipped with a LAB6 source at 120 kV by using a Gatan UltraScan charge-coupled device camera.

### Monolayer tension measurements

Tension measurements were performed using a drop tensiometer device (TRACKER; Teclis-IT Concept; Ben M’barek et al., 2017). The principle of the drop profile analysis is based on the determination of the shape of a LD suspended in another liquid form from a video image and its comparison with theoretical profiles calculated from the Gauss-Laplace equation. The RP (MilliporeSigma; R-1512) drop (neutral lipid phase), containing the DOPC phospholipid or not, was formed in buffer (50 mM Hepes, 120 mM potassium acetate, 1 mM magnesium chloride, pH 7.4) at RT. The tension was allowed to stabilize for a few minutes (it decreases by the continuous absorption of phospholipids to the oil–water interface). Then, the drop is compressed by decreasing its volume until complete saturation of the interface is reached (marked by a plateau of tension during compression).

### DIB experiments

The DIB experiments were performed following a previous study (Ben M’barek et al., 2017). An oil phase containing phospholipids was prepared first. DOPC, dioleoyl phosphatidylethanolamine, and dioleoyl phosphatidic acid were purchased from Avanti Polar Lipids. Lipids were mixed to the RP (MilliporeSigma; R-1512) oil at a final lipid concentration of 0.2% wt/wt. (∼10% chloroform was in the final mixture in the case of DOPC initial stabilization of the DIBs, and we let it evaporate over time; for the other phospholipids, chloroform was evaporated before addition of RP.) Then, an emulsion was prepared by mixing the buffer with RP (1:5 vol/vol). Finally, the same volumes of emulsion and phospholipids in RP were put together, and the resulting emulsion was placed on a hydrophobic coverslip.

### Interfacial tension measurements

A pendant droplet tensiometer designed by Teclis Instruments was used to measure the interfacial tension of oil–water interfaces. All experiments were conducted at RT. To create oil–buffer interfaces, oil drops (10 µl) were formed at the tip of a J-needle submerged in 5 ml HKM buffer (50 mM Hepes, 120 mM potassium acetate, and 1 mM MgCl_2_ at pH 7.4).

### Statistical analyses

All figures were processed in Inkscape version 0.92.2. Bar plots represent means ± SD or SEM as indicated in the figure legends. Statistical significance was determined by two-tailed Welch’s or Student’s *t* tests or by Mann-Whitney tests, as indicated. P values <0.05 were considered statistically significant.

### Online supplemental material

Fig. S1 shows quantification of RE content. Fig. S2 shows LD formation in the absence of preexisting LDs. Fig. S3 shows neutral lipids in cells with or without seipin. Videos 1 and 2 are 3D reconstructions of LDs in LRAT-GFP. Table S1 lists *S. cerevisiae* strains and plasmids used in this study. Table S2 lists quantitative PCR primers used in this study.

## Supplementary Material

Table S1 lists *S. cerevisiae* stains and plasmids used in this study.

Table S2 lists quantitative PCR primers used in this study.
