# Supplementary material for: Retinyl esters form lipid droplets independently of triacylglycerol and seipin
Source: J Cell Biol. 2021 Jul 29;220(10):e202011071. doi: 10.1083/jcb.202011071 (PMC8327380; doi:10.1083/jcb.202011071)
Supplement: Table S1 — lists S. cerevisiae stains and plasmids used in this study. [file JCB_202011071_TableS1.docx]

**Table S1. *S. cerevisiae* stains and plasmids used in this study**

| **strain/plasmid** | **genotype or description** | **source** |
| --- | --- | --- |
| BY4741 | MATa *his3∆1 leu2∆0 met15∆0 ura3∆0* | Laboatory collection |
| BY4742 | Mat α *his3*Δ1 *leu*2Δ0 *lys*2Δ5 *ura*3Δ0 | laboratory collection |
| VCY63 | BY4742 Mat α *his3Δ1 leu2Δ0 lys2Δ5 ura3Δ0 ERG6-mCherry::HIS3* | this study |
| VCY64 | BY4742 *MATα his3∆1 leu2∆0 lys2∆0 ura3∆0 met15∆0, are1::KanMX, are2∆::KanMX, trp1, lro1::TRP1 dga1::lox ERG6-mCherry::HIS3* | this study |
| KKY067 | BY4742 Mat α *his3Δ1 leu2Δ0 lys2Δ5 ura3Δ0 ERG6-mCherry::HIS3 p415GPD-LRAT-LEU2* | this study |
| KKY068 | BY4742 *MATα his3∆1 leu2∆0 lys2∆0 ura3∆0 met15∆0, are1::KanMX, are2∆::KanMX, trp1, lro1::TRP1dga1::lox ERG6-mCherry::HIS3 p415GPD-LRAT-LEU2* | this study |
| NOY53 | BY4741 MATa *his3Δ1 leu2Δ0 met15Δ0 ura3Δ0 scs3::HIS3 yft2::KanMX* | Choudhary *et al.*, 2015 |
| KKY160 | BY4742 *MATα his3∆1 leu2∆0 lys2∆0 ura3∆0 met15∆0, are1::KanMX, are2∆::KanMX, trp1, TRP1::GAL-LRO1 dga1::lox ERG6-mCherry::HIS3* | this study |
| KKY184 | BY4742 *MATα his3∆1 leu2∆0 lys2∆0 ura3∆0 met15∆0, are1::lox, HIS3-GAL-ARE2, trp1, lro1::KanMX, dga1::lox, ERG6-mCherry::URA3* | this study |
| KKY186 | BY4742 *MATα his3∆1 leu2∆0 lys2∆0 ura3∆0 met15∆0, are1::KanMX, are2∆::KanMX, trp1, TRP1::GAL-LRO1 dga1::lox ERG6-mCherry::HIS3, sei1::CloNAT* | this study |
| KKY183 | BY4742 *MATα his3∆1 leu2∆0 lys2∆0 ura3∆0 met15∆0, are1::lox, HIS3-GAL-ARE2, trp1, lro1::KanMX, dga1::lox, ERG6-mCherry::URA3, sei1::CloNAT* | this study |
| KKY168 | BY4742 *MATα his3∆1 leu2∆0 lys2∆0 ura3∆0 met15∆0, are1::KanMX, are2∆::KanMX, trp1, lro1::TRP1dga1::lox ERG6-mCherry::HIS3, sei1::HYG, p415GPD-LRAT-LEU2* | this study |
| KKY072 | BY4742 *MATα his3∆1 leu2∆0 lys2∆0 ura3∆0 met15∆0, are1::KanMX, are2∆::KanMX, trp1, TRP1::GAL-LRO1 dga1::lox, sei1::Ca-URA3* | this study |
| KKY181 | BY4742 *MATα his3∆1 leu2∆0 lys2∆0 ura3∆0 met15∆0, are1::lox, HIS3-GAL-ARE2, trp1, lro1::KanMX, dga1::lox, sei1::CloNAT* | this study |
| KKY069 | BY4742 *MATα his3∆1 leu2∆0 lys2∆0 ura3∆0 met15∆0, are1::KanMX, are2∆::KanMX, trp1, lro1::TRP1dga1::lox, p415GPD-LRAT-LEU2, sei1::HIS3* | this study |
| KKY166 | BY4742 *MATα his3∆1 leu2∆0 lys2∆0 ura3∆0 met15∆0, are1::KanMX, are2∆::KanMX, trp1, TRP1::GAL-LRO1 dga1::lox ERG6-mCherry::HIS3, SEI1-GFP-URA3* | this study |
| KKY216 | BY4742 *MATα his3∆1 leu2∆0 lys2∆0 ura3∆0 met15∆0, are1::lox, HIS3-GAL-ARE2, trp1, lro1::KanMX, dga1::lox, SEI1-GFP-URA3, Ycplac111-ERG6-mCherry::LEU2* | this study |
| KKY169 | BY4742 *MATα his3∆1 leu2∆0 lys2∆0 ura3∆0 met15∆0, are1::KanMX, are2∆::KanMX, trp1, lro1::TRP1dga1::lox ERG6-mCherry::HIS3 p415GPD-LRAT-LEU2, SEI1-GFP-URA3* | this study |
| KKY232 | BY4742 *MATα his3∆1 leu2∆0 lys2∆0 ura3∆0 met15∆0, are1::KanMX, are2∆::KanMX, trp1, TRP1::GAL-LRO1 dga1::lox ERG6-mCherry::HIS3, sei1::CloNAT Ycplac111-NVJ1(1-260)-SEI1-GFP-LEU2* | this study |
| KKY234 | BY4742 *MATα his3∆1 leu2∆0 lys2∆0 ura3∆0 met15∆0, are1::lox, HIS3-GAL-ARE2, trp1, lro1::KanMX, dga1::lox, ERG6-mCherry::URA3, sei1::CloNAT Ycplac111-NVJ1(1-260)-SEI1-GFP-LEU2* | this study |
| KKY233 | BY4742 *MATα his3∆1 leu2∆0 lys2∆0 ura3∆0 met15∆0, are1::KanMX, are2∆::KanMX, trp1, lro1::TRP1dga1::lox ERG6-mCherry::HIS3, sei1::HYG, p415GPD-LRAT-LEU2, Ycplac33-NVJ1(1-260)-SEI1-GFP-URA3* | this study |
| KKY238 | BY4742 *MATα his3∆1 leu2∆0 lys2∆0 ura3∆0 met15∆0, are1::KanMX, are2∆::KanMX, trp1, TRP1::GAL-LRO1 dga1::lox ERG6-mCherry::HIS3, sei1::CloNAT, p415GPD-LRAT-LEU2, Ycplac33-NVJ1(1-260)-SEI1-GFP-URA3* | this study |
| KKY080 | Mat α *his3*Δ1 *leu*2Δ0 *lys*2Δ5 *ura*3Δ0 p415GPD-*LRAT-GFP-LEU2*, Ycplac33-SEC63-mCherry-URA3 | this study |
| KKY081 | BY4742 *MATα his3∆1 leu2∆0 lys2∆0 ura3∆0 met15∆0, are1::KanMX, are2∆::KanMX, trp1, lro1::TRP1dga1::lox ERG6-mCherry::HIS3, p415GPD-LRAT-GFP-LEU2, Ycplac33-SEC63-mCherry-URA3* | this study |
| KKY178 | BY4742 *MATα his3∆1 leu2∆0 lys2∆0 ura3∆0 met15∆0, are1::KanMX, are2∆::KanMX, trp1, TRP1::GAL-LRO1 dga1::lox ERG6-mCherry::HIS3, NEM1-yeGFPx3-URA3* | this study |
| KKY217 | BY4742 *MATα his3∆1 leu2∆0 lys2∆0 ura3∆0 met15∆0, are1::lox, HIS3-GAL-ARE2, trp1, lro1::KanMX, dga1::lox, Ycplac111-ERG6-mCherry::LEU2, NEM1-yeGFPx3-URA3* | this study |
| KKY179 | BY4742 *MATα his3∆1 leu2∆0 lys2∆0 ura3∆0 met15∆0, are1::KanMX, are2∆::KanMX, trp1, lro1::TRP1dga1::lox ERG6-mCherry::HIS3 p415GPD-LRAT-LEU2, NEM1-yeGFPx3-URA3* | this study |
| KKY177 | BY4742 *MATα his3∆1 leu2∆0 lys2∆0 ura3∆0 met15∆0, are1::KanMX, are2∆::KanMX, trp1, TRP1::GAL-LRO1 dga1::lox ERG6-GFP::HIS3, PEX30-mCherryx2::URA3* | this study |
| KKY222 | BY4742 *MATα his3∆1 leu2∆0 lys2∆0 ura3∆0 met15∆0, are1::lox, HIS3-GAL-ARE2, trp1, lro1::KanMX, dga1::lox, Ycplac111-ERG6-mCherry::LEU2,, PEX30-mCherryx2::URA3* | this study |
| KKY176 | BY4742 *MATα his3∆1 leu2∆0 lys2∆0 ura3∆0 met15∆0, are1::KanMX, are2∆::KanMX, trp1, lro1::TRP1dga1::lox ERG6-GFP::HIS3 p415GPD-LRAT-LEU2, PEX30-mCherryx2::URA3* | this study |
| pKKE018 | p415GPD-LRAT *(LEU2/CEN*), full length LRAT expressed by the *GPD1* promoter | This study |
| pKKE020 | p415GPD-LRAT-GFP *(LEU2/CEN*), full length LRAT-GFP expressed by the *GPD1* promoter | This study |
| pAT124 | YCplac33-*SEC63*-mCherry (*URA3/CEN*), Sec63-mCherry expressed by the *SEC63* promoter | This study |
| pKKE42 | YCplac33-*SEI1-GFP* (*URA3/CEN*), Sei1-GFP expressed by the *GPD1* promoter | This study |
| pKKE40 | YCplac111-NVJ1-SEI1-GFP (*LEU2/CEN*), Nvj1(1-260-Sei1-GFP expressed by the *GPD1* promoter | This study |
