# Supplementary material for: Retinyl esters form lipid droplets independently of triacylglycerol and seipin
Source: J Cell Biol. 2021 Jul 29;220(10):e202011071. doi: 10.1083/jcb.202011071 (PMC8327380; doi:10.1083/jcb.202011071)
Supplement: Table S2 — lists quantitative PCR primers used in this study. [file JCB_202011071_TableS2.docx]

**Table S2. Quantitative PCR primers used in this study.**

| **gene** | **species** | | **source** | | | |
| --- | --- | --- | --- | --- | --- | --- |
| Lrat | Mus musculus | | Tuohetahuntila *et al.*, 2017 | | | |
| Ywhaz | Mus musculus | | Tuohetahuntila *et al.*, 2017 | | | |
| Hmbs | Mus musculus | | Tuohetahuntila *et al.*, 2017 | | | |
| Hprt | Mus musculus | | Tuohetahuntila *et al.*, 2017 | | | |
| Gapdh | Mus musculus | | this study | | | |
|  | | **primer** | | | **5'-sequence-3'** | **T_m_ (^o^C)** |
|  | | F | | | GAA GGT CGG TGT GAA CGG | 61 |
|  | | R | | | TGA AGG GGT CGT TGA TGG |  |
| Actb | Mus musculus | | | this study | | |
|  | | **primer** | | | **5'-sequence-3'** | **T_m_ (^o^C)** |
|  | | F | | | AGC TCC TTC GTT GCC GGT CCA | 57 |
|  | | R | | | TTT GCA CAT GCC GGA GCC GTT G |  |
| Rps18 | Mus musculus | | this study | | | |
|  | | **primer** | | | **5'-sequence-3'** | **T_m_ (^o^C)** |
|  | | F | | | GAT CCC TGA GAA GTT CCA GCA C | 57 |
|  | | R | | | ACC ACA TGA GCA TAT CTC CGC |  |
